# MEASURING QUALITY IN LONG-TERM SERVICES AND SUPPORTS THROUGH PERSON-REPORTED OUTCOMES: VALUE OF NCI DATA

**DOI:** 10.1093/geroni/igad104.0805

**Published:** 2023-12-21

**Authors:** Tetyana Shippee, Stephanie Giordano

## Abstract

Long-Term Services and Supports (LTSS) include medical, social, and personal care services that people may need to perform activities of daily living due to physical or cognitive impairments. Measuring the quality of LTSS is essential for improving the care and outcomes of people with disabilities and older adults and ensuring that services meet consumer needs. Several frameworks have been developed to measure LTSS quality, including the National Quality Forum’s LTSS Framework. Yet, few data sources are available to measure person-reported quality in HCBS. This symposium will provide an overview of National Core Indicators (NCI) as a unique data source to address this gap. It also includes presentations from researchers from organizations in two states who utilize NCI data – Minnesota and Massachusetts – who will share their perspectives about the value of the data, results from empirical analyses, and how they use and plan to use the data to inform policy. Researchers from the Lurie Institute for Disability Policy will present findings on the association between person-centered planning and key person-reported outcomes in Medicaid HCBS. Researchers from the University of Minnesota School of Public Health and Brown University will share findings from national NCI data on unmet needs among older adults by dementia status and race/ethnicity. They will also share results of analyses in MN, linking person-reported HCBS plan quality and healthcare utilization, using claims data. Collectively, this symposium will address the gap in measurement of HCBS quality nationally and identify key directions for policy and future research.

